# “食品安全分离分析-高分辨技术专辑”引言

**DOI:** 10.3724/SP.J.1123.2023.10031

**Published:** 2023-11-08

**Authors:** Feng ZHANG

## 引言

食品安全事关人民群众身体健康和生命安全,事关经济安全与社会和谐稳定。高分辨分离分析技术是保障食品安全的重要手段,食品基质复杂,目标物种类繁多,且含量水平低,加之近年来采用结构修饰合成的新型非食用物质、新型毒素等未知有害物造成的食品安全事件时有发生,如何快速准确侦查食品中未知有害物成为现代食品安全分析的一大挑战。

近年来,随着色谱、质谱、信息技术等学科领域的不断进步,新型分离分析技术、质谱在线联用技术及微型装置等不断涌现,为食品安全检测技术能力的提升带来新的机遇。科研工作者开发了系列原位、实时、在线、无损、高通量、可视化的色谱分离分析技术,极大地推动了食品检验学的发展。

鉴于此,我们组织了“食品安全分离分析”系列的第二个专辑——高分辨技术专辑,诚挚邀请了国内食品安全高分辨分离分析研究与应用相关的高等院校、科研院所研究人员为本专辑撰稿。 经过专家严格评审,最终接受视角1篇、研究论文7篇和技术与应用2篇,涵盖液相色谱-四极杆/轨道阱/飞行时间质谱、气相色谱-四极杆/飞行时间质谱等高分辨技术在食品安全中的应用。最终10篇文章以专辑形式刊出,以期探讨并展示该领域的前沿技术及其进展。希望本专辑能为读者带来启迪,碰撞出智慧的火花,不断提升我国食品安全检验检测技术的研究水平。

衷心感谢本专辑作者以及审稿专家的大力支持和倾情奉献!

本专辑客座主编

中国检验检疫科学研究院

张 峰 研究员


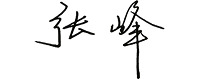


2023年10月25日

